# Experience in the Child/Youth Mental Health Centre of Leganés (Madrid) during the first lockdown

**DOI:** 10.1192/j.eurpsy.2022.703

**Published:** 2022-09-01

**Authors:** B. Díez Valle, S. De La Fuente Cabrero, A. Martín Roldán, C. Zurita Zarco

**Affiliations:** 1 Hospital Universitario Severo Ochoa, Psychiatry, Leganés, Spain; 2 Addiction Centre for Youth Los Mesejo, Psychology, Madrid, Spain; 3 Child/Youth Mental Health Centre of Leganés, Psychology, Leganés, Spain; 4 Hospital General La Mancha Centro, Psychiatry, Alcázar de San Juan, Spain

**Keywords:** Covid-19 pandemic, childhood mental health, lockdown, behavioural disturbances

## Abstract

**Introduction:**

The municipality of Leganés has been very vulnerable to the effects of the crisis derived from the COVID-19 pandemic (both due to the incidence of the infection and the socioeconomic situation). Multiple studies show that children and adolescents, especially those with a psychiatric background, have been one of the most affected groups during the confinement.

**Objectives:**

Firstly, to describe the characteristics of clinical care at the Child/Youth Mental Health Centre of Leganés (Madrid) during the first lockdown (March-June 2020). Secondly, to present data on the clinical evolution of the patients along this period (n = 720).

**Methods:**

Descriptive study and literature review.

**Results:**

**Figure:**
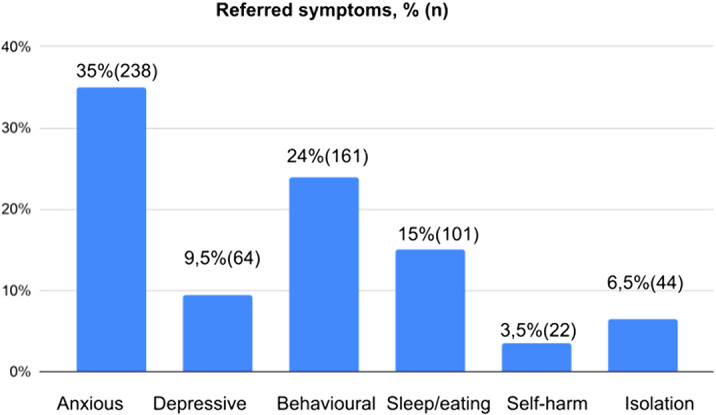

Clinical care during the period of strict confinement was mainly by telephone, although the most serious cases were attended in person. In addition, referral to Intensive outpatient programs was interrupted. The results show 56% of patients remained stable. Anxious symptoms (35%) and behavioural disturbances (24%) were most frequently referred (Figure 1), It is noteworthy that the most critical cases (such as suicide attempts or domestic violence) were observed in adolescents and that at least 11% of patients increased their use of electronic devices.

**Conclusions:**

The first confinement stage was particularly stressful for families and especially for children and adolescents, although most patients remained psychopathologically stable. However, other studies have found a significant increase in child and adolescent mental health problems during the following months (de-escalation stage).

**Disclosure:**

No significant relationships.

